# Strong Geometrical Effects in Submillimeter Selective Area Growth and Light Extraction of GaN Light Emitting Diodes on Sapphire

**DOI:** 10.1038/srep17314

**Published:** 2015-11-27

**Authors:** Atsunori Tanaka, Renjie Chen, Katherine L. Jungjohann, Shadi A. Dayeh

**Affiliations:** 1 Materials Science Program, University of California San Diego, La Jolla CA, 92093, USA; 2 Department of Electrical and Computer Engineering, University of California San Diego, La Jolla CA, 92093, USA; 3 Center for Integrated Nanotechnologies, Sandia National Laboratories, Albuquerque NM 87185, USA

## Abstract

Advanced semiconductor devices often utilize structural and geometrical effects to tailor their characteristics and improve their performance. We report here detailed understanding of such geometrical effects in the epitaxial selective area growth of GaN on sapphire substrates and utilize them to enhance light extraction from GaN light emitting diodes. Systematic size and spacing effects were performed side-by-side on a single 2” sapphire substrate to minimize experimental sampling errors for a set of 144 pattern arrays with circular mask opening windows in SiO_2_. We show that the mask opening diameter leads to as much as 4 times increase in the thickness of the grown layers for 20 μm spacings and that spacing effects can lead to as much as 3 times increase in thickness for a 350 μm dot diameter. We observed that the facet evolution in comparison with extracted Ga adatom diffusion lengths directly influences the vertical and lateral overgrowth rates and can be controlled with pattern geometry. Such control over the facet development led to 2.5 times stronger electroluminescence characteristics from well-faceted GaN/InGaN multiple quantum well LEDs compared to non-faceted structures.

The selective area growth (SAG) of III-V compound semiconductor materials has been studied for decades because of its numerous advantages in controlling the growth structure and morphology^1^,^2^,^3^,^4^,^5^,^6^. Most notably, SAG allows the reduction of threading dislocations at the grown surface by trapping and bending with lateral overgrowth^5^,^7^,^8^,^9^,^10^,^11^,^12^ and it allows accommodation of thermal stresses during heteroepitaxial growth^13^,^14^. The SAG of arsenide and phosphide III-V materials has been analyzed quite extensively but less studies were reported for nitride materials except for nano-scale mask openings and spacings^6^,^7^,^9^,^15^,^16^,^17^,^18^,^19^,^20^,^21^. The interest in submillimeter scale heteroepitaxy and SAG have witnessed recently increased interest for large scale integration of light emitting diodes (LEDs)^22^,^23^,^24^ and the development of high power devices^25^,^26^,^27^,^28^,^29^,^30^,^31^. It is therefore timely to conduct detailed and systematic studies of the SAG of GaN in submillimeter scale mask openings. The deep understanding of geometric effects for SAG is necessary for the application to versatile devices, particularly when device scaling and their array density become relevant.

In this work, we conducted SAG GaN in oxide masks in previously unexplored geometries of circular openings with 20 μm to 450 μm diameters and edge-to-edge spacings on a 2” c-plane sapphire wafer (See Supplementary Fig. S1). With systematic observation by scanning electron microscopy (SEM) and thickness profilometry, we characterized the SAG GaN on sapphire both qualitatively and quantitatively using mass-transport limited growth models. To shed light on the SAG morphology control that is developed in this study, we fabricated GaN/InGaN quantum well LEDs and demonstrated 2.5 times enhanced light extraction with carefully engineered structures.

## Results and Discussion

### Generic geometric effects in SAG GaN on c-sapphire

#### Systematic observation of the SAG GaN with different geometries

**Figure 1:**
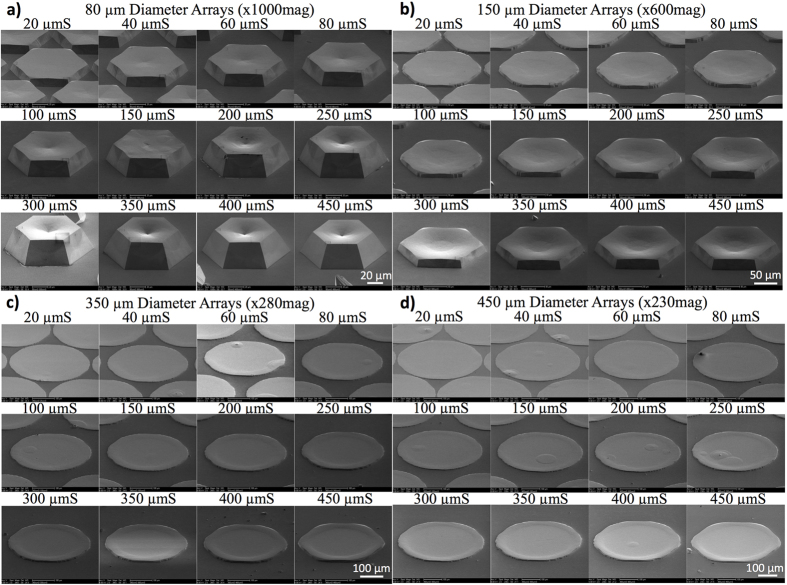
45°-angled SEM images of the SAG GaN structures for different mask openings (**a**) 80 μm, (**b**) 150 μm, (**c**) 350 μm, and (**d**) 450 μm for edge-to-edge spacings in the range of 20 μm to 450 μm. Strong vertical growth rate enhancement was observed for all diameters and spacings.

We first systematically characterized the SAG GaN with different mask openings and spacings by glanced angle SEM as shown in Fig. 1. The 45° angled-view SEM images show strong size and spacing dependence of the vertical growth rate manifesting clear geometrical effects in the SAG GaN on sapphire at such submillimeter scales. For a given opening diameter, the overall height of the GaN structure was increased with increasing mask spacing accompanied by a significant increase in the dot edge height. A concave shaped surface morphology evolved as the mask spacing was increased. The dot edges always exhibited larger heights than the center indicating more adatom arrival and incorporation at the mask edges. Similarly, for a given spacing, the heights of the SAG GaN structure decreased with increasing the mask diameter.

**Figure 2:**
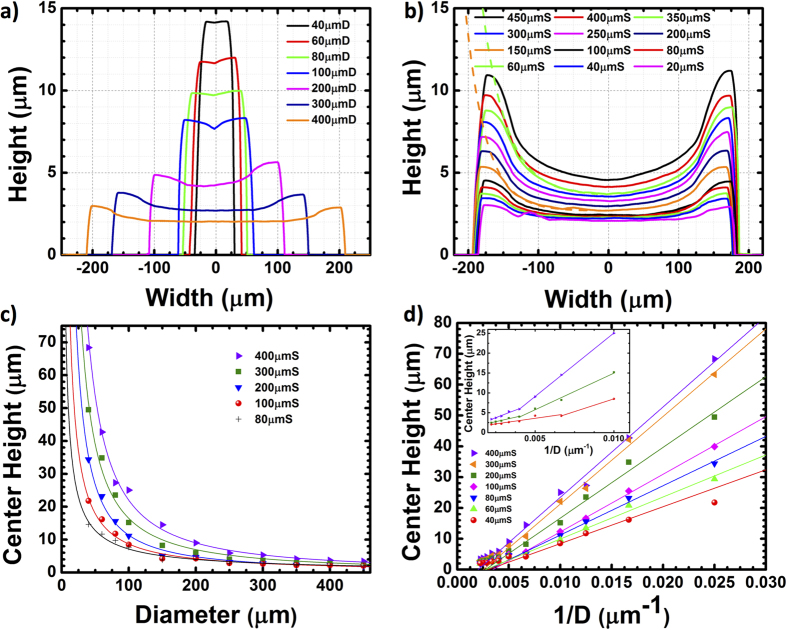
Surface profilometry on the grown structure with (**a**) fixed spacing at 20 μm and (**b**) fixed diameter at 350 μm. The solid lines represent the measured thickness profile and dashed lines are fitted lines for 350 μmS and 150 μmS dots to extract the Ga adatom diffusion length. (**c**) The heights of the grown structures as a function of the mask diameters and (**d**) the inverse of the mask diameters. The inset is a blow up of the large diameter region of the plot.

For quantitative comparison, the height of the grown structures were measured by surface profilometry and plotted with respect to mask dot diameters and spacings in Fig. 2. To eliminate pattern array edge effects, we chose for our analysis the center dot from each of the 5 × 5 hexagonal array patterns and measured the dot height profile along with <1
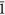
00> direction. From the fixed spacing data (Fig. 2a) we observed a remarkable growth rate difference for different dot diameters. The center height of the 40 μm diameter (referenced thereafter as μmD) dot was found to be 4 times taller than that of the 400 μmD at 20 μm spacing (referenced thereof as μmS). Edge effects become prominent at larger dot diameters (and larger spacings as discussed later). From Fig. 2a, the edge height for the 400 μmD is twice as high as its center. For sufficiently small diameters (<80 μm), the edge effects are generally minimal and become visible for larger spacing as can be observed in Fig. 1a. For dot diameters larger than 60 μm, concave-shaped surface morphology was observed as shown for the 150 μmD in Fig 1b. The flatness of the surface was improved for larger diameter dots as shown in Fig. 1c,d.

#### Ga adatom diffusion length on GaN surface

The above observations can be explained by the relative scales of pattern radii and the Ga adatom surface diffusion length on the c-plane GaN dot surface. If the Ga diffusion length is larger than the dot radius, the Ga adatom adsorbing/arriving at the edge of the dot can reach the center of the dot leading to a nearly flat dot surface. In contrast, if the Ga diffusion length is smaller than the radius of the dot, Ga adatoms adsorbed/arrived at the dot edge cannot make it to the dot center, resulting in nonuniformity of the dot height across its diameter. If the dot radius is much larger than the Ga adatom diffusion length, the height gradient will be limited to the edges of the dot that extend over a distance that is comparable to the Ga adatom diffusion length on the c-plane GaN surface, and the center region will be nearly flat. To extract the Ga adatom surface diffusion length, we utilize the formulation developed by Rozhavskaya *et al.* who considered the edge effects on the growth rate of GaN stripes and extracted Ga adatom diffusion length to be in the range of 5 μm to 24 μm^32^. In the 1D approximation along the diameter of a GaN dot, the solutions for the diffusion equation accounting for impinging flux and desorption of Ga adatoms from the GaN surface together with the two boundary conditions of a constant Ga adatom density at the dot center and zero density at the dot edge yield^32^:


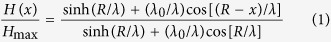


here, *R* is the original mask dot radius, 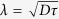 is the incorporation-limited diffusion length of Ga adatoms on the GaN surface and *λ*_0_ is the diffusion length on the dot side facets. For simplicity in the extraction of the diffusion lengths, we assume that the usually different diffusion length on different planes to be identical on our GaN dot top surface and sidewalls (*λ *= *λ*_0_). The measured plots with fitting curves using equation (1) are shown in Fig. 2b with corresponding extracted values listed in Table 1 to be 29 μm–35 μm. The extracted diffusion length has implications on the synergetic effects during the GaN SAG on c-Al_2_O_3_. When the spacings are the same but diameters are different, the smaller diameter dot has a higher diffusion length. When the diameters are the same but the spacing increases the diffusion length correspondingly becomes higher. In our experiment, the growth conditions are the same such that the input molar fraction of precursors is identical. Thus, the difference in the diffusion length results from the mask geometry itself rather than the growth conditions as observed by Rozhavskaya *et al*^32^. By changing the mask geometry, the local V/III ratio at the mask edge can be tailored. For larger spacings, the larger collection area of Ga adatoms on the SiO_2_ mask leads to more Ga diffusion to the growth interface and the effective V/III ratio at the mask edge decreases. Similarly for smaller diameters, the relative ratio of the dot area to the collection area on SiO_2_ is smaller resulting in enhanced Ga concentration at the edge and lower effective V/III ratio. Naturally, it is more likely for Ga adatoms to react with NH_3_ when the V/III ratio is higher, which decreases the Ga adatom diffusion length. These trends agree well with those previously observed in the SAG InGaAs growth^33^.

The surface nonuniformity became significant when the diameter of the openings were equivalent to or larger than the diffusion length of Ga on GaN surface. Therefore, this edge enhanced growth effect is specific to SAG in wide patterns. The thickness variation throughout the structure will affect the material thickness in critical regions of devices, such as in quantum wells, which can be detrimental to their performance and should be suppressed. Based on these observations, wider mask openings and tight edge-to-edge spacings would be the best-suited SAG geometries that can be utilized for attaining uniform device morphologies.

#### Size dependent vertical growth rate

To quantitatively analyze the size-dependent vertical growth rates in SAG GaN on sapphire, we need to fully account for reactant direct impingement as well as their mass transport on both the SiO_2_ and the GaN surfaces to the growth interface. This is customary for whisker^34^ and nanowire^35^,^36^ growth and has been employed recently in the GaN SAG^37^. The reactant impingements on the top surface of the opened GaN surface, *J*_*top*_, surface diffusion of the reactants on SiO_2_, *λ*_*sub*_, surface diffusion of the reactants on GaN sidewall, *λ*_*sw*_, and gas phase diffusion flux from the substrate and impingement on the sidewall surface, *J*_*sw*_, all contribute to SAG GaN growth rate^37^. It is also important to note that the SAG occurs simultaneously normal to and parallel to the substrate surface, typically referred to as vertical growth and lateral overgrowth, respectively. For same diameter areas, the increase of lateral overgrowth is relatively small and can therefore be ignored. The lateral overgrowth rate as a function of diameter and spacing is discussed later With these considerations, the growth rate in SAG can be written as^37^:


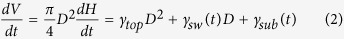


where *V* is the volume of the GaN dot, *H* is the height, *D* is the diameter, *γ*_*top*_ is a time independent constant related to top surface impingement, and *γ*_*sw*_ and *γ*_*sub*_ are time dependent impingement and adsorption parameters and are constant in our case due to the fixed growth time of 1 hour. *γ*_*sw*_ in our experiment is however dependent on facet development which varies with size and spacing as deduced from Fig. 1. Therefore, the vertical growth rate dependency should be characterized with the following equation:


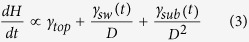


In Fig. 2c,d, we plotted the SAG GaN dot center height as a function of mask opening diameter and inverse diameter, respectively. The fitted lines in Fig. 2c are proportional to 1/*D*^*β*^ where 1 ≤ *β* ≤ 2. Figure 2d shows further details of the diameter dependence and its inset highlights the large diameter region where we see a transition from *β *= 1 for small diameters to *β *= 2 for large diameters. From Equation (3), we can deduce that the sidewall diffusion coefficient *γ*_*sw*_ dependence dominates the SAG dot growth height. The larger spacing dots had heights with larger slopes (*β *= 1) as a function of inverse diameter because the diffusion of Ga is more favorable as shown in Table 1. The systematic SEM images in Fig. 1 demonstrated that the growth facets for larger dot diameters were not well developed whereas the growth facets for smaller dot diameters were well developed for all spacings. The non-flat and not well-developed facets impede Ga adatom diffusion on the sidewall and slow down the increase in the vertical height with inverse diameter whereas well-developed facets led to an increased slope of the height with inverse diameter.

#### Spacing dependent vertical growth rate

**Figure 3:**
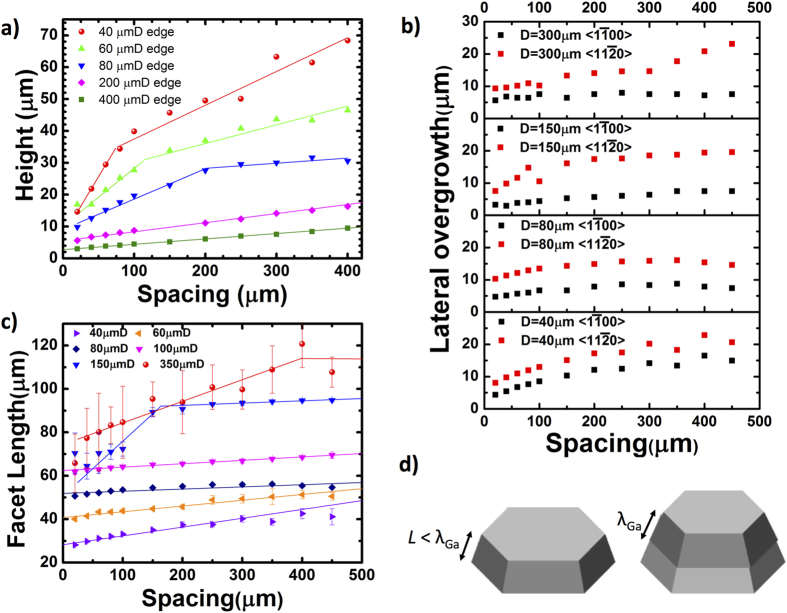
(**a**) Edge heights for the grown structures. (**b**) The lateral overgrowth lengths and (**c**) the facet lengths as a function of the mask spacings. The facet lengths are defined as the average length of the six sides of the grown hexagon base. (**d**) Schematic illustration of the collection area contributing to the vertical growth when the sidewall length is less than or equivalent to Ga diffusion length on GaN (left) and when it is larger than the diffusion length (right).

In addition to the strong size effects, the array edge-to-edge spacing was found to have significant influence on the vertical growth rate of SAG GaN as illustrated in Fig. 3a. The smaller diameter dots exhibited two different slopes with spacing whereas the height for the larger diameter dots showed weaker yet a fixed linear increase with spacing. This can also be explained by the relative difference between the Ga diffusion length which we extracted to be ~30 μm on the GaN surface and the GaN dot height. When the thickness is less than 30 μm, the collection area of SAG GaN structure increased with spacings because most of the impinging Ga adatoms on the GaN dot sidewall can diffuse on the sidewall and reach the top GaN growth interface. However, when the thickness becomes larger than 30 μm, the collection area of SAG GaN structure remains fixed within 30 μm from the top surface. The fixed collection area leads to an overall reduction of the rate of increase of the height with spacing than for thinner dots. This also corroborates with our earlier discussions that for smaller dot diameters, the Ga adatom surface diffusion lengths are high due to a lower effective V/III ratio at the dot edges. This size enhanced growth rate for smaller diameters diminishes when the collection area becomes constant (Fig. 3d) leading to a transition from sharp increase of growth height with spacing to slower increase at larger spacings when the dot height exceeded 25–30 μm.

#### Spacing dependent lateral growth rate, facet length and facet evolution

**Figure 4:**
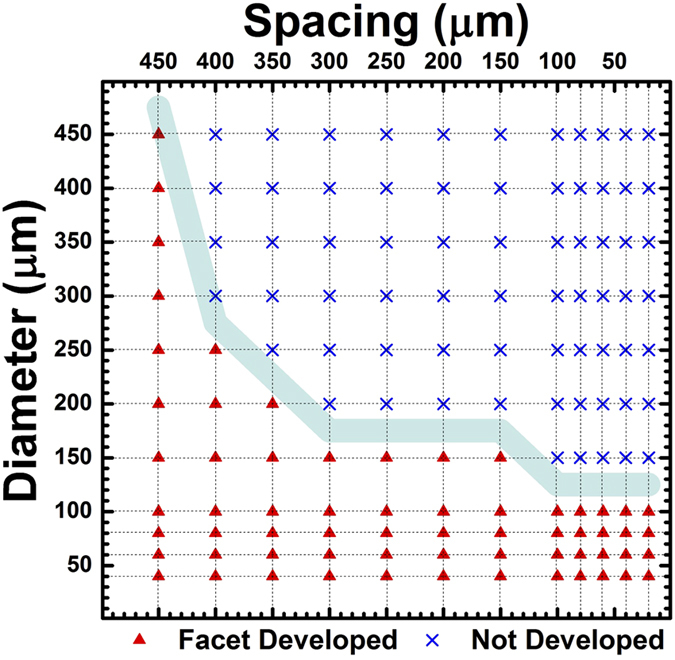
Facet evolution with the mask geometry as was observed by SEM. The triangles represent the grown structure with six complete well-developed facets and the crosses represent the grown structure with at least one rough sidewall.

Not only the vertical growth rate but also lateral overgrowth rate depends on the spacing. Figure 3b shows the lateral overgrowth length as a function of the mask spacing. The lateral overgrowth length was determined by subtracting the original circular opening from the total length for each of <1
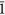
00> and <11
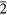
0> directions of the SAG GaN. As can be seen in Fig. 3b, the lateral overgrowth length depends on the growth direction. The growth in <1
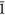
00> direction usually has less lateral length than that in the <11
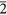
0> direction. The {1
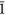
01} facets have low surface energies^38^,^39^ and the GaN circular dot transforms its shape to a hexagonal dot to reduce the total surface energy by developing these {1
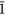
01} facets that exhibit lower growth rate. Therefore, in order to understand the lateral overgrowth rate, we need to take into consideration the facet formation. Figure 3c shows a plot of the facet length as a function of the mask spacing. We define the “facet length” as the average length of the six sides of the grown hexagon that were measured by SEM. For smaller diameters, the change of the facet length with spacing is weak and can be fitted with a single line. In contrast, for larger diameters, the facet length increases sharply with spacing and then plateaus for larger spacings. The slopes in the plateau region for larger diameter and larger spacing are similar to those for the smaller diameter. This can be explained with the facet development as a function of diameter and spacing that was characterized with top-view SEM (see Supplementary Fig. S3 online) and plotted in Fig. 4. The facet was defined as “developed” for structures that had no rough surface and with six complete and sharp facets. From the figure, we can find that there are well-defined transition points from well developed to non-developed facets which corroborate with the transition points from non saturated facet growth to saturated facet growth in Fig. 3b,c. For instance, the facet of the sample with a 150 μmD developed for spacings larger than 150 μm from Fig. 4 and in Fig. 1b, and the slope of <11
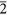
0> lateral overgrowth changed at the spacing 150 μm in Fig. 3b. Additionally, smaller diameter dots which already had developed complete facets for all spacing as listed in Fig. 4 exhibited a fixed and small slope for the facet length as a function of spacing in Fig. 3c. From these observations, we can conclude that the facet length growth rate in the <1
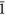
00> decreased after the formation of a well-developed {1
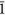
01} facets and increased rapidly prior to the formation of well-developed facets.

#### Time dependent facet evolution

The time evolution for facet formation is shown in Supplementary Fig. S3 where three different samples were grown for 2 min, 4 min and 6 min. As in prior studies, we focused on only the center dot of the array, here for 80 μmD and 150 μmS sample. For the 2 min growth time, the facets didn’t develop and the shape of the SAG GaN was circular similar to the starting original mask shape. For the 4 min growth time, the facets started to develop by growing faster in the <11
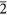
0> direction but the sidewall surface of the facets was still rough. For 6 min growth time, the facet was completely developed by forming smooth {1
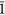
01} facets for this mask geometry. After the facet development, all the six facets had the same length. This indicated that the facet evolution can be tuned by controlling the growth time as well as the mask geometry.

### Enhancement of LED light extraction by tailoring array pattern structure

**Figure 5:**
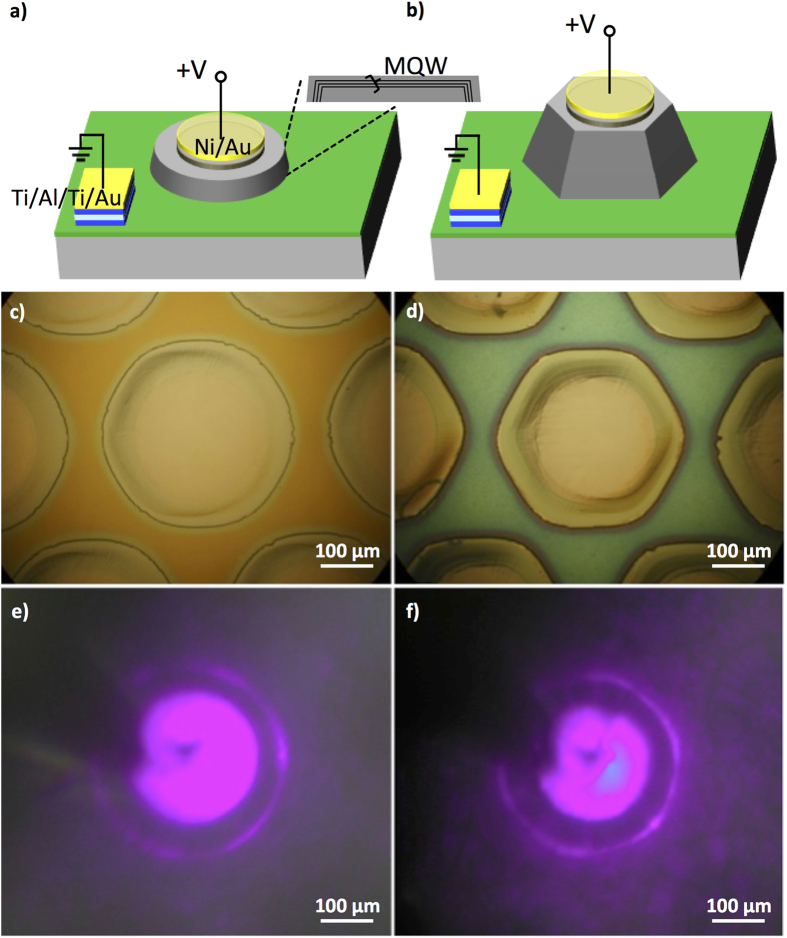
Schematic illustration of the device structure for (**a**) non-faceted SAG LED and (**b**) well-faceted SAG LED. Optical microscope images of (**c**) non-faceted and (**d**) well-faceted and their emitting images in (**e,f**) respectively.

From these growth studies, we developed the knowledge to tailor the SAG morphology by changing either mask opening size, spacing or growth time. These morphological changes are expected to influence the performance of devices made from these structures. To this end, we fabricated InGaN/GaN multiple quantum well (MQW) LED structures with/without well-developed facets as shown in Fig. 5a,b. To develop the well-faceted and non-faceted structures and yet maintain the same MQW layer thicknesses, we controlled the growth time for a pre-MQW n+ SAG GaN layer for 45 min (non-faceted) and 2.5 hours (well-faceted) as shown in the optical microscope images of Fig. 5c,d. A semi-transparent contact of Ni 5 nm/Au 5 nm was deposited atop of the dot followed by annealing in 100 sccm oxygen flow at 550 °C for 10 min which result in ohmic-like characteristics^40^,^41^,^42^,^43^. Ti 30 nm/Al 70 nm/Ti 10 nm/Au 50 nm ohmic contact was deposited on the n+ GaN bottom layer after etching of SiO_2_. Their current voltage characteristics are shown in Supplementary Fig. S4. Electroluminescence characterization was performed on both well-faceted and non-faceted LED structures.

The optical microscope images of the well-faceted and non-faceted LED samples at the drive current of 10 mA are shown in Fig. 5e,f, respectively. We observed strong emission through the thin metal contacts and some light reflection on the sidewalls. From the emitting images in Fig. 5e,f, relatively broadened light reflection and more light transmission parallel to the substrate surface were observed for the non-faceted samples while the reflected light of the well-faceted sample was sharper, less transmissive and more intense.

**Figure 6:**
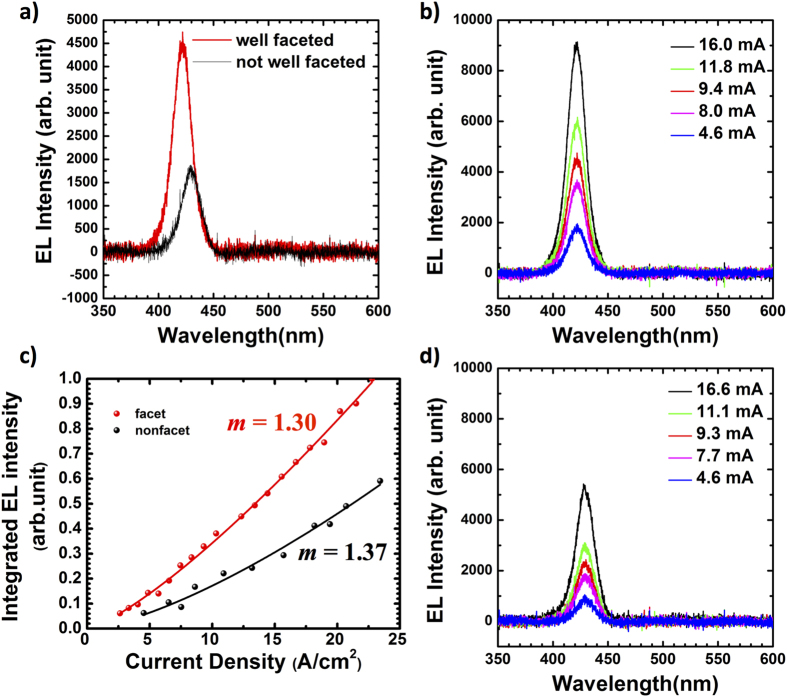
(**a**) Electroluminescence of the SAG LED samples with/without well-developed facets at 10 mA injection current. Electroluminescence of (**b**) well-faceted LED and (**d**) non-faceted LED with different currents. (**c**) Integrated EL intensities of well-faceted/non-facet LED with different current densities.

**Figure 7:**
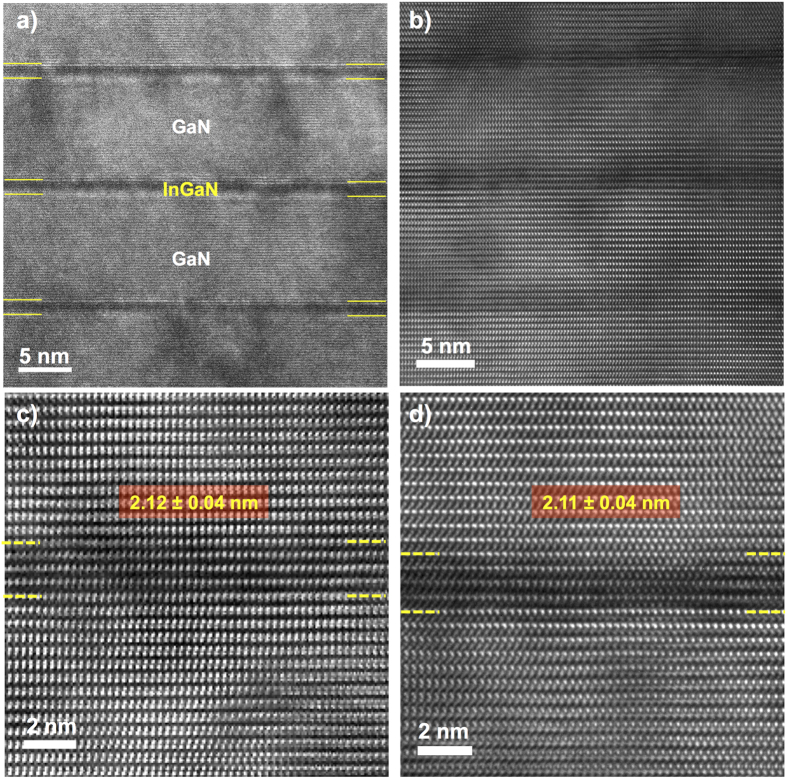
Cross-sectional TEM images of (a,c) well-faceted and (b,d) non-faceted MQW structures near the center of the structures. (**a,b**) show the uniform growth of three cycles of InGaN MQWs. (**c,d**) are zoomed-in images of (**a,b**), respectively.

The electroluminescence (EL) data is shown in Fig. 6 for quantitative comparison of the light intensities. A well resolved InGaN/GaN MQW emission peak was observed for both of the well-faceted and non-faceted LED devices and the full width at half maximum of the spectra were found to be ~20 nm for both devices. It is well known that GaN LEDs grown on semipolar substrates or surfaces exhibits stronger light emission due to polarization field reduction and consequent stronger spatial overlap in the electron and hole wavefunctions^44^,^45^,^46^,^47^. In this work, we deposited metals on the c-plane top-surface such that the current spreading layer and consequently light emission is mainly from the polar surface. Despite this, Fig. 6a shows that the well-faceted LED had ~2.5 times higher EL peak intensity compared to the non-faceted LED. In addition, the EL peak of the well-faceted LED was at 421 nm which is ~9 nm blue-shift compared to that of the non-faceted LED. The EL peak wavelength can depend on MQW thickness, strain, drive current, temperature, and In concentration^48^,^49^,^50^,^51^. To identify the origin of the observed blue-shift, we investigated the MQW structures by high resolution transmission electron microscopy (HRTEM). Figure 7 presents the TEM characterization of the well-faceted and non-faceted structures at the center of each structure. We found that the MQWs of both structures have similar QW thicknesses of 2.1 nm and QB thicknesses of 8.6 nm at the center of the structure (Supplementary Table S1). Therefore, we can conclude that the peak shift can be caused by the In concentration difference between the well-faceted and non-faceted samples. It is known that In incorporation efficiencies on different GaN surface planes are different^44^,^52^,^53^,^54^,^55^. T. Wunder *et al.* reported a 50% higher indium incorporation for {1
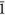
01} semipolar facets in comparison to c-plane growth^44^. In our structures, In adatoms diffusing from the mask were trapped more on the semipolar facets on than the c-plane top surface. Furthermore, as previously discussed for Ga adatom diffusion effects on the SAG growth rate, less In adatoms can reach the top of the SAG structure for the dots having thicker sidewalls than the In diffusion length. From the peak shift, we estimate around 14% lower In composition in the faceted LED when compared to the non-faceted LED. Figure 6b,d show EL spectra of the well-faceted and non-faceted LEDs for different injection currents. The dispersion of the EL intensities with different currents was larger for the faceted LED. This implies that the sharp facets act as mirrors with strong index difference that reflect light to be emitted normal to the LED instead of diffracting it randomly in the lateral direction at the rough LED surface for the non-faceted LEDs.

To infer the dominant recombination mechanism, we plot in Fig. 6c the *L-J* curves which are characterized by


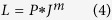


where *L* is the integrated EL intensity, *J* is the current density (A/cm^2^), *P* is a constant and *m* is an exponent parameter^56^,^57^. The exponent *m* can be used to characterize the emission mechanism of the SAG LED^57^. In our case of *L-J* dependence, both of the devices showed a super linear dependence (*m *~ 1.3), which implies that most injected carriers recombine radiatively. With higher injection currents, Auger processes would become dominant with lower *m* values, a regime we didn’t access in our experiments because of current limitation of our measurement setup. This result indicates that the well developed semipolar facets are not only enhancing light emission by reducing polarization electric field but also assist in better light extraction in the c-axis direction.

## Conclusion

To summarize, we showed in this study strong mask opening size and spacing effects in the GaN SAG on sapphire and demonstrated that such effects have implications on LED performance. By tailoring the SAG mask opening diameter, we observed as much as 4 times increase in the vertical height for 20 μm spacings. By changing the SAG spacing, we observed as much as 3 times increase in the vertical height at a dot diameter of 350 μm. We extracted the Ga adatom diffusion lengths to be ~29–35 μm by fitting the surface profile plots, and attributed this difference to the effective V/III ratio or different mask geometries which also controlled the SAG surface morphology. The quantitative analysis of the mask opening size and spacing dependence on the vertical and lateral growth rate indicates that facet evolution of the SAG structure is a significant factor to determine the growth rate. With such understanding of and control over the growth morphology, we demonstrated that well-faceted structures exhibited stronger electroluminescence than non-faceted structures at the same current density. The strong morphology-performance effects in the LED structures developed in this work pave the way for higher efficiency LEDs.

### Experimental Section

For the growth of SAG GaN films, we utilized a 3 × 2” Thomas Swan/Axitron close-coupled showerhead (CCS) metal-organic chemical vapor deposition (MOCVD) system with trimethylgallium (TMGa) and ammonia precursors, and H_2_ carrier gas. To circumvent surface preparation effects on nucleation in different size/spacing patterns, we first grew a 1 μm thick GaN buffer layer on a single 2 inch c-Al_2_O_3_ wafer. A 200 nm of SiO_2_ was then deposited on the wafer surface by plasma enhanced chemical vapor deposition (PECVD) and conventional photolithography then followed in order to pattern 144 different hexagonal arrays of circular openings, which consist of combinations of 12 different mask openings and edge-to-edge spacings (see Supplementary Fig. S1 online). The mask openings and spacings in SiO_2_ were varied from 20 μm to 450 μm, and were etched by a diluted buffered oxide etch (BOE) with a 6:1 volume ratio of 40% NH_4_OH in water to 49% HF in water. The exposed GaN surface was treated with hydrochloric acid (HCl) solution (36–38%) at 60 °C for 3 min in order to remove native gallium oxides (Ga_x_O_y_)^58^. We carried out the SAG on these wafers at a temperature of 1050 °C which was calibrated at the susceptor surface, and a chamber pressure of 100 mbar for 1 hour. A V/III ratio of 2250 was employed for the SAG, which corresponds to a planar growth rate of 1 μm/hr on 2” c-Al_2_O_3_ wafer. This mask pattern allowed us to eliminate sample-to-sample fluctuations of the growth and consider only geometrical effects. The morphologies of the grown structures were characterized by scanning electron microscope (SEM) and Dektak surface profilometer. In addition, the growth for different times with fixed growth conditions was conducted to observe facet evolution with time.

Post growth rate, dopant, and metal-contact optimization, and to demonstrate the engineering aspects of our geometric SAG studies, we tailored the growth structure to control the morphologies of InGaN/GaN multiple quantum well (MQW) blue LEDs that exhibited greater light extraction effects. Their electroluminescence characteristics were determined using a DU420A-OE Andor charge coupled device (CCD) camera mounted on an Oriel instrument cornerstone 260 motorized 1/4 m monochromator and using a National Instruments interface control board with Labview automated measurements. The HRTEM characterization were performed in an FEI Tecnai F30 300 kV microscope for the well-faceted and non-faceted samples for investigating the MQW structures. The locations for the cross-sectional TEM images and the TEM images near the edge of the structures are shown in Supplementary Fig. S5 and the MQW thicknesses at different points were summarized in Supplementary Table S1.

## Additional Information

**How to cite this article**: Tanaka, A. *et al.* Strong Geometrical Effects in Submillimeter Selective Area Growth and Light Extraction of GaN Light Emitting Diodes on Sapphire. *Sci. Rep.* **5**, 17314; doi: 10.1038/srep17314 (2015).

## Supplementary Material

Supplementary Information

## Figures and Tables

**Table 1:** Diffusion lengths for selected different mask geometries.

Mask geometry	Extracted diffusion length (μm)
350 μmD 150 μmS	29
350 μmD 350 μmS	33
80 μmD 350 μmS	35
