# Supplementary material for: Strong Geometrical Effects in Submillimeter Selective Area Growth and Light Extraction of GaN Light Emitting Diodes on Sapphire
Source: Sci Rep. 2015 Nov 27;5:17314. doi: 10.1038/srep17314 (PMC4661445; doi:10.1038/srep17314)
Supplement: Supplementary Information [file srep17314-s1.pdf]

Strong Geometrical Effects in Submillimeter Selective Area Growth and Light Extraction of GaN Light Emitting Diodes on Sapphire

Atsunori Tanaka[1], Renjie Chen[2], Katherine L. Jungjohann[3], Shadi A. Dayeh[1,2,*]

[1] Materials Science Program, University of California San Diego, La Jolla CA, 92093, USA
[2] Department of Electrical and Computer Engineering, University of California San Diego, La Jolla CA, 92093, USA
[3] Center for Integrated Nanotechnologies, Sandia National Laboratories, Albuquerque NM 87185, USA
[*]sdayeh@ece.ucsd.edu

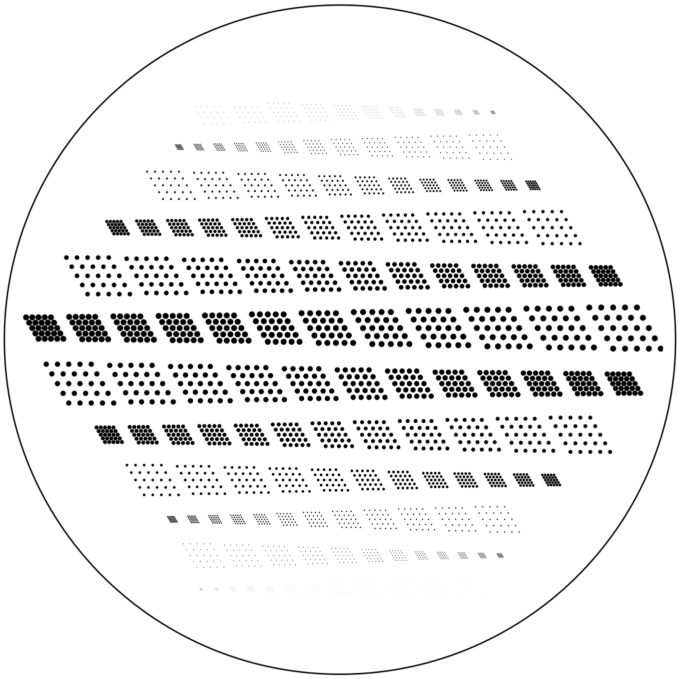

Figure S1. Photomask design for SAG GaN on 2 inch wafer. These arrays of circular patterns consist of 12 different edge-to-edge spacings for each of 12 different diameter dots, resulting in 144 different array patterns in total. This photomask can fit in a single 2 inch wafer, which allows us to minimize experimental sampling errors.

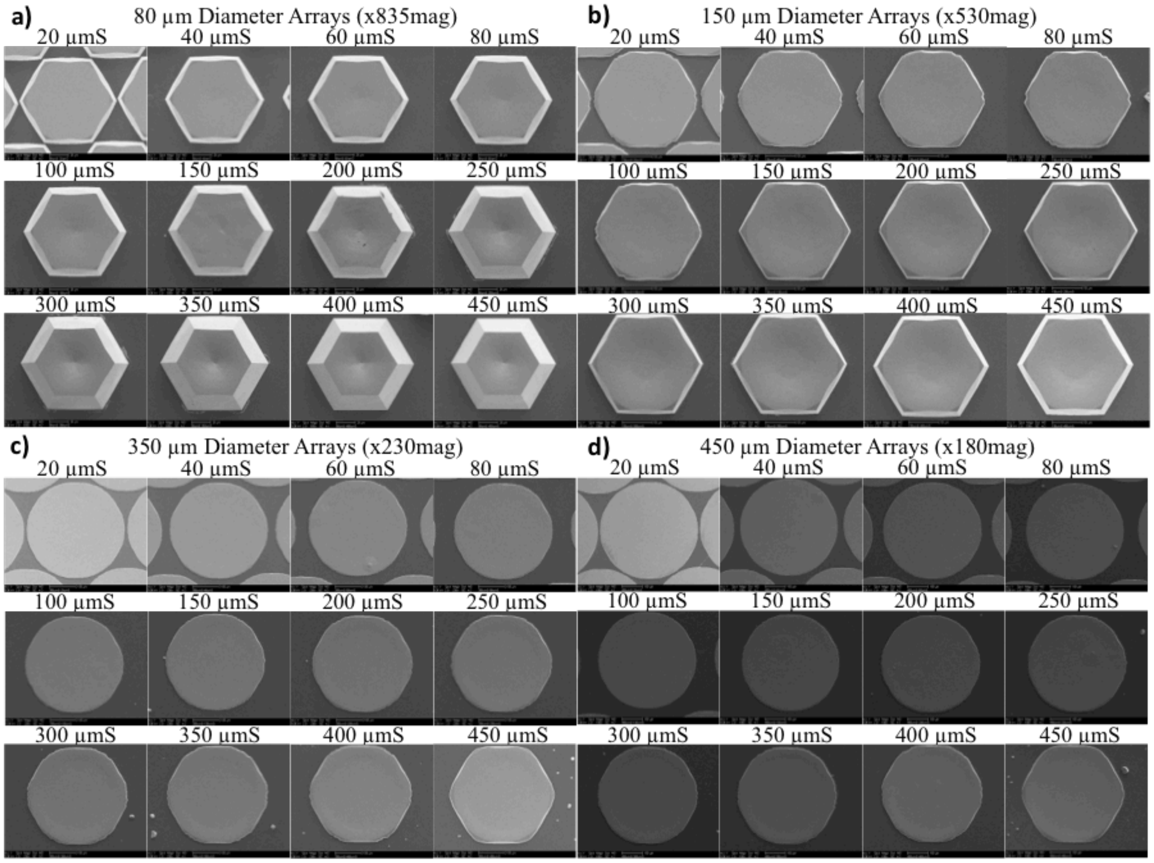

Figure S2. Top view SEM images of the SAG GaN structures for different mask openings (a) 80 µm, (b) 150 µm, (c) 350 µm, and (d) 450 µm for edge-to-edge spacings in the range of 20 µm to 450 µm.

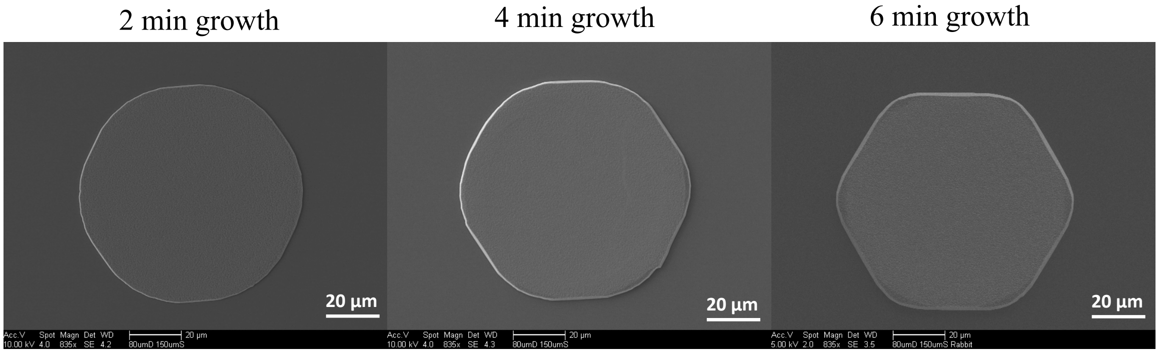

Figure S3. Top-view SEM image of the samples with 80 µmD and 150 µmS with different growth time.

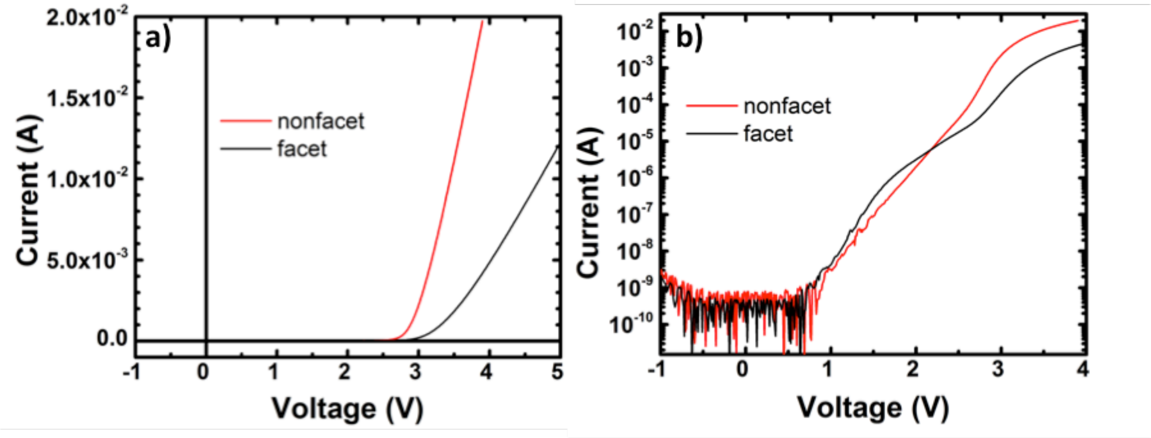

Figure S4. Current-voltage characteristics of (red line) non-faceted LED and (black line) well-faceted LED with (a) linear scale and (b) log scale.

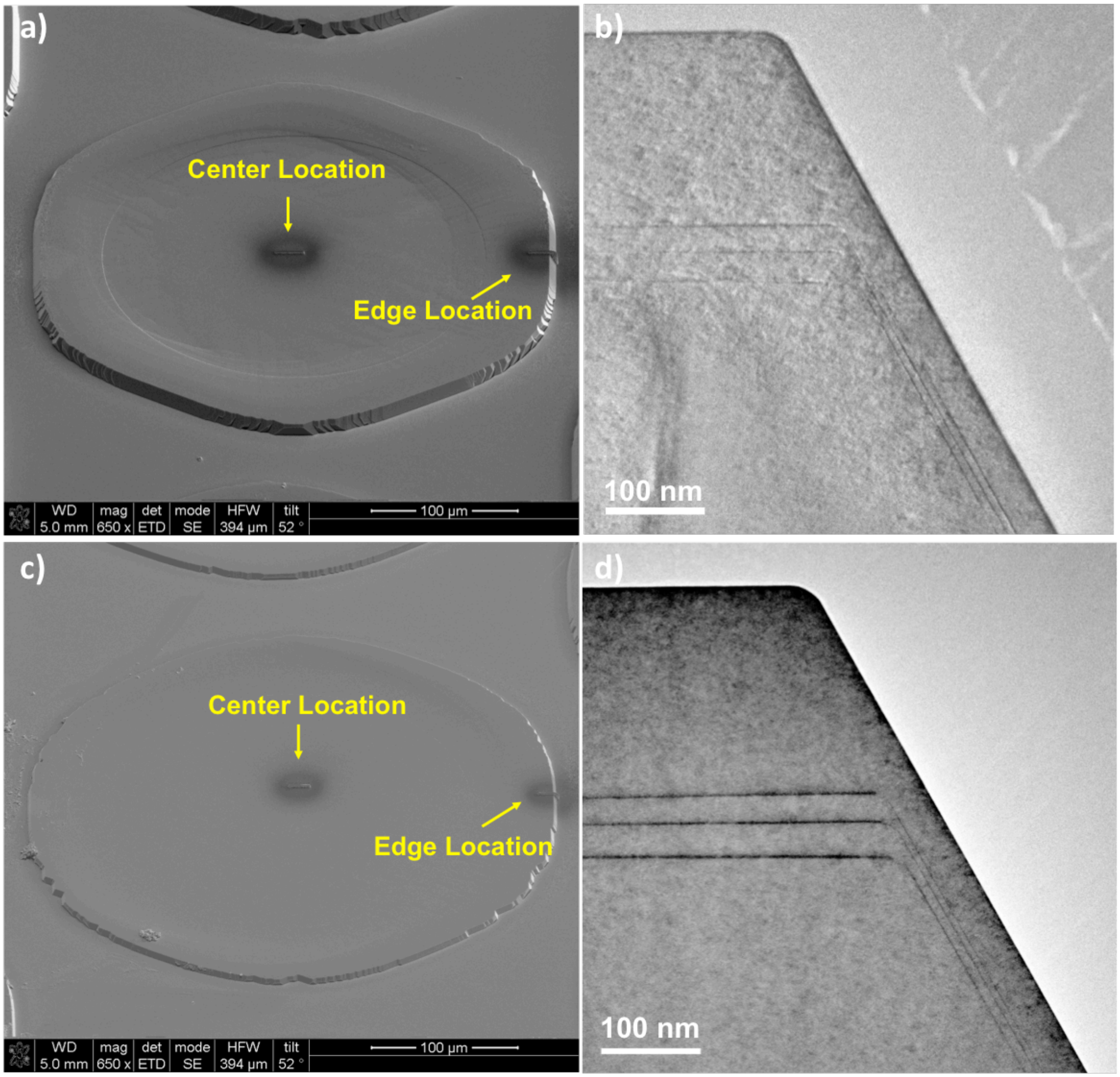

Figure S5. Angled view SEM images of (a) well-faceted and (c) non-faceted samples and their cross-sectional cut areas. The cross-sectional HRTEM images at the edge locations of (b) well-faceted and (d) non-faceted samples.

Sample location		QB thickness (nm)	QW thickness (nm)
Non-Faceted	Center	8.56 ± 0.21	2.11 ± 0.04
	Edge	27.78 ± 2.98	3.01 ± 0.41
	Sidewall	6.76 ± 2.01	1.42 ± 0.36
Well-Faceted	Center	8.58 ± 0.27	2.12 ± 0.04
	Edge	14.95 ± 0.81	2.07 ± 0.25
	Sidewall	7.35 ± 0.48	1.24 ± 0.01

Table S1. MQW thicknesses of the well-faceted and non-faceted LED at different points of the structures; center, edge and sidewall of the structure. These thicknesses were measured from TEM images and averaged over 10 lines for each point.
